# Children's internal migration and subjective wellbeing of older parents left behind: Spiritual or financial support?

**DOI:** 10.3389/fpubh.2023.1111288

**Published:** 2023-04-03

**Authors:** Ying Lu, Yi-Yang Lin, Jun-Qiao Qu, Yi Zeng, Wan-Zong Wu

**Affiliations:** ^1^School of Public Administration, Zhongnan University of Economics and Law, Wuhan, China; ^2^Business School, Yangzhou University, Yangzhou, China

**Keywords:** subjective wellbeing, internal migration, parents left behind, spiritual support, financial support

## Abstract

**Introduction:**

Against the background of population aging and large-scale internal migration, this study uses an ordered logit with two-way fixed effects to examine the effect of children's internal migration on the subjective wellbeing of parents left behind. The study is based on the China Family Panel Studies database.

**Methods:**

Data were obtained from CFPS (China Family Panel Studies), and ordered logit with two-way fixed effects was used to test the total effect of children's internal migration on subjective wellbeing of parents left behind, and KHB test was used to separate intergenerational spiritual support and intergenerational financial support to examine the intergenerational support preferences of parents left behind.

**Results:**

The results show that children's internal migration has a significant negative effect on the subjective wellbeing of parents left behind, mainly through the reduction of intergenerational spiritual support. Furthermore, intergenerational financial support significantly mitigates this negative effect. There is heterogeneity in the direction of the total wellbeing effect across parents' preferences, as well as in the masking effect of financial support. However, the effect of financial support never fully offsets the effect of spiritual support.

**Discussion:**

To cope with the negative effects of children's internal migration on parents, positive measures should be taken to change parental preferences.

## 1. Introduction

One-third of the world's population is on the move and migrating today, with profound effects on social order (1). The data of the seventh census shows that among China's 1.4 billion people, the population separated from households is 493 million, of which the migrant population accounts for around 376 million–26.86% of the country's total population. This is an increase of nearly 70% from 2010. Thus, the phenomenon of large-scale population migration has become an important issue for social development. From the perspective of an aging population, the proportion of people aged 60 years or older was 18.70% in 2020, 5.44% higher than that in 2010. This statistic has obvious urban-rural differences, with 23.81% of the older adult population located in rural areas and 15.82% in urban areas. The root cause of the serious aging of the rural population is the imbalance in the migrant population's age structure due to the outflow of young laborers while older adults and children are left behind.

The movement of young labor occurs not only between rural and urban areas, but also between cities and towns, and is a universal phenomenon. Owing to the constraints of China's household registration system and objective conditions, it is not common for older people to move with their children to the cities where they work. According to a 2015 survey by the National Health and Family Planning Commission, the proportion of the migrant population of older adults aged 60 years and above was 7.2%. Only 25% of the total older adult population indicated the main purpose as a family reunion and aging in a different place. Against the backdrop of the dual realities of domestic mass migration and further aging, the phenomenon of family separation is bound to persist and continue to grow.

According to the economics of happiness, social development is as much about achieving wealth growth as it is about achieving human happiness, and the economics of happiness paradigm calls for maximizing human wellbeing as the ultimate goal. Economic development is an important means of enhancing the wellbeing of the population, and enhancing people's wellbeing is the fundamental purpose of economic development. China's economy has been greatly enhanced in the past four decades. However, is the wellbeing of the large group of older parents left behind under population migration being satisfied? An analysis of this issue is very relevant in the current situation.

Wellbeing can be measured along two dimensions. One is evaluative wellbeing, which refers to a person's overall evaluation of the quality of his or her life based on job satisfaction, life satisfaction, financial satisfaction, and so on. This measurement provides a comprehensive and objective assessment of a person's wellbeing. The other is experienced wellbeing, which is also referred to as subjective wellbeing (SWB). This is people's overall emotional and cognitive evaluation of their life experiences, which captures the positive and negative emotions they experience from moment to moment (2). Through these two dimensions, scholars have extensively explored the factors influencing the wellbeing of older adults, such as health status (2, 3), activity participation choices (4), health insurance (5), physical activity (6), volunteering activity (7), and divergent thinking (8), for example. Many studies have focused on the relationship between income and wellbeing. For instance, a significant positive relationship was found between household income and wellbeing, while an increase in the average income in the city where one lives significantly reduces wellbeing to the extent that it counteracts the wellbeing effect of an increase in absolute income (9). SWB has also been delineated as emotional wellbeing and life satisfaction (10). In this regard, it was found that higher income leads to higher life satisfaction, but it does not increase emotional wellbeing. However, low income was associated with both low emotional wellbeing and low life satisfaction (10). Note though that some scholars are skeptical. For example, using logistic regression models, Shah et al. (11) found no significant empirical association between household income and wellbeing.

Extensive research has shown the important influence of social support and social connectedness on the wellbeing of older adults. Social support has been positively associated with emotional wellbeing and psychological satisfaction and significantly impacts the overall SWB (12). Furthermore, a significant positive relationship has been found between indicators of various dimensions of social support and wellbeing (13). Therefore, the quality and quantity of social support can be an appropriate determinant and predictor of wellbeing among older adults (13). In other research, 389 rural older adults in the Maule region of central Chile were studied to estimate an ordered logistic regression model (14). The study found that the frequency of meals with others was significantly associated with wellbeing (14). Using three measures of wellbeing—hedonic, eudaimonic, and evaluative—others found that negative perceptions of neighborhoods as unsafe, unattractive, and feeling isolated were associated with poor wellbeing (15). Supporting the idea of dissociative effects, a case study in the UK confirmed that social class and social mobility had a negative impact on SWB, as older adults lost their connection to their class of origin (16). In addition, self-efficacy has been confirmed as a full mediator of social support affecting mental wellbeing, predicting wellbeing through perceived social support, which was moderated by optimism and increasing happiness (17). Finally, social phobia, anxiety, depression, and debilitation significantly reduce wellbeing among older adults. Despite this, social phobia in the older adult population has thus far not been given due attention (18).

Of the types of social support, support from family, especially intergenerational support from children, has a significant impact on older adults. While they can turn to different sources of support when they need it, including neighbors, friends, and professionals, the primacy of family members as a source of support for older adults is self-evident, since it is reflected favorably in national legal provisions (19). Among the African American older population, a significant positive effect was found in informal social support from family, such as frequency of family contact, family closeness, and life satisfaction (20). Furthermore, estimating the causal effect of intergenerational cohabitation on parents' SWB confirmed that older adults living with adult children were happier than those without such living arrangements (21). It was also found that in urban areas, geographical proximity to adult children was inversely *U*-shaped in relation to older parents' wellbeing. Older adults who lived independently and in closer proximity to their adult children had significantly higher life satisfaction than those who lived with their children or at a greater distance from their children (22). In addition, data from 34 European countries used to compare indicators of wellbeing among single widowed women indicated that widows with children were happier than those without who usually lived alone (23). Finally, findings also suggest that intergenerational social support, self-esteem, and loneliness are significantly related to SWB. Here, structural equation modeling demonstrated that self-esteem and loneliness partially mediate the effect of intergenerational social support on SWB (24).

Under the influence of Chinese Confucianism, East Asian countries have converged on a regional identity and consciousness that actively advocate filial piety. It is this understanding of filial piety among older adults that make intergenerational support from children particularly important. A regression analysis based on an assessment of six domains of filial piety—respect, happiness, care, greeting, obedience, and support—showed that lower scores on filial expectations significantly increased the risk of depressive symptoms among older adults and that these expectations may be related to SWB (25). It was also found that interaction with children was useful in improving older parents' wellbeing and that designing and implementing programs to connect with children could help improve the happiness, joy, and satisfaction of older adults in institutions (26). Also important is that China is transitioning from a child-centered to a parent-centered approach, which has increased the wellbeing of rural older adults by 28% (27). A study on urban retirees aged 60 years and above in China indicated that receiving financial and spiritual support from their children increased life satisfaction overall. Furthermore, living with children positively impacted the life satisfaction of single retirees (28). Another some scholars hold the skeptical view, arguing that “raising children against aging,” a core idea of Confucianism, is no longer applicable to older adults in China, and living with children and receiving material transfers or support from them have no significant effect on older parents' wellbeing (29).

Some scholars focused on the impact of migration on the family members left behind. Migration can be either international or internal. Academic attention tends to focus on international migration. In this regard, data from Moldova, which has the highest migration rate in the world, confirmed the positive effect of migration on BMI, mobility, and self-rated health among the older adults left behind (30). Similarly, a study in Turkey found that older adult migrants may gain from migration, which was reflected in their life satisfaction (31). Estimation of a series of SWB functions also confirmed a positive relative remittance effect on rural individuals and households. Here, the wellbeing of rural households increased because of the remittances from migrating family members (32).

However, scholarly findings also support the negative effect of migration on the older adults left behind. For example, a causal link has been suggested between the deteriorating health outcomes of older Mexican parents left behind and the children of immigrants in the United States (33). Other findings confirm the negative impact of children migrating on the health of parents left behind (34, 35). From the perspective of intergenerational family systems, one study showed that the negative emotional effects of separation from family were prevalent among parents left behind. These emotional effects included sadness, longing, guilt, and worry (36). Migration imposes an emotional cost on those left behind, which adversely affects their emotional health (37). While migration remittances can compensate for the partial loss of the self-reported health and mental health of parents left behind, they do not do so for the deterioration of other health indicators (38). Actually, family member migration abroad has been associated with increased stress and depression, which were not offset by remittances (39). A random survey of more than 400 households in three municipalities in the central part of the state of Cochabamba, Bolivia, found that economic benefits from international migration increased levels of household wellbeing; however, household wellbeing declined even more due to the family disintegration caused by migration. Ultimately, migrating households were significantly less happy than non-migrating households (40).

Based on the literature review, fewer studies have focused on the impact of internal migration on the SWB of the older parents left behind than those investigating international migration. This may be because of the greater spatial extent of migration, longer periods of family separation, and more pronounced impact caused by international migration. However, the scale of internal migration in China, which has a massive population and vast territory, has reached alarming levels. In China, internal migration is also large in scope, long in duration, and deep in impact. Despite this, research is lacking on the impact of social support and the magnitude of spiritual and financial support. Here, children's migration has a clear impact on parents left behind through intergenerational transfer.

On the one hand, provincial data on the migrant population show that provinces with negative net migration are mostly economically underdeveloped, suggesting that money is a major driver of the outflow of young labor. In turn, children's internal migration can improve households' economic strength and provide financial support for parents. On the other hand, as the size of Chinese households has decreased from 3.10 people in 2010 to 2.62 people in 2020,^1^ the support of empty nesters has become a social problem that cannot be ignored. Furthermore, children's internal migration will reduce the time available to provide life care for older adults, thereby decreasing parents' quality of life by causing illness and producing symptoms such as mental loneliness. Confucian culture is accustomed to using the family as the basic unit to build a network of relationships with other social actors outwardly; therefore, family stability is a necessary condition for social stability and national prosperity. In the context of the reality that family support still bears a key task to provide for older adults, the purpose of this study is to explore the impact of the internal migration of children on the SWB of parents left behind, which has led to the spatial separation of families.

## 2. Material and methods

### 2.1. Setting and participants

The data in this study were derived from the China Family Panel Studies (CFPS) micro-database. The CFPS data used a three-stage sampling method with implicit stratification to obtain an equal probability sample, thus ensuring the representativeness and validity of the data. The CFPS data from 2014 and before lacked the question related to “relationship with children” and could not be matched with children's codes. Thus, CFPS2016 and CFPS2018 were selected to construct the panel data analyzed in this study.

### 2.2. Measurements

#### 2.2.1. Explained variable

The explained variable in this study was parents' SWB. Respondents' SWB values were extracted from the question “How happy do you feel” in the “subjective attitude” module of the CFPS database. Respondents chose any integer value from 0 to 10 to evaluate their SWB: the higher the value, the higher the SWB. The study population was restricted to older adults aged 60 years and above with children, following the World Health Organization's definition of older adults as those aged 60 years and above.

#### 2.2.2. Explanatory variable

The core explanatory variable in this study was “children's internal migration.” The number of children migrating within the country was counted and a binary variable was generated. At least one child migrating within the country was assigned a value of 1 and no child migrating within the country, a value of 0. A valid sample size of 12,927 was obtained by matching the codes of outworkers in the CFPS database with those of children in the individual self-response questionnaire.

#### 2.2.3. Control variables

This study controlled for the following demographic and economic characteristics of the research objective: gender (female = 0, male = 1), age, marriage (unmarried = 1, married = 2, cohabiting = 3, divorced = 4, widowed = 5), education (based on the highest education completed by the respondent: illiterate/semi-literate = 1, primary school = 2, junior high school = 3, high school/junior high school/technical school/vocational high school = 4, college = 5, bachelor's degree = 6, and master's degree = 7), household registration (non-agricultural household registration = 0; agricultural household registration = 1), health status (any integer value from 1 to 5, with higher values representing poorer health), social status (any integer value from 1 to 5, with higher values representing higher social status), personal income (respondent's total income from work in the past 12 months), and per capita household expenditure (total expenditure of respondent's household in the past 12 months/number of people in the household).

## 3. Models

### 3.1. Theoretical model

Children's internal migration often results from certain needs and positive expectations such as improved social status and economic power, which have a positive effect on the SWB of older parents through intergenerational transfer. Children's internal migration increases the geospatial distance between them and their parents, resulting in insufficient daily care, affection, and spiritual comfort, all of which have a negative effect on the SWB of older people. The ultimate direction of the effect of children's internal migration on parents' SWB depends on the relative magnitude of these two effects. In this study, we simplified the positive effect of financial support and the negative effect of spiritual support.^2^ Since both effects are provided by children, assuming there was saturation in the support that children could provide as the supply side, then according to the production possibility boundary curve:


(1) $$\begin{array}{lll} mI^{2}+nT^{2} & = & \omega \end{array}$$

(2) $$\begin{array}{lll} I & = & P_{x}x \end{array}$$

(3) $$\begin{array}{lll} T & = & qt \end{array}$$

(4) $$\begin{array}{lll} U & = & x^{\alpha }t^{\beta } \end{array}$$

where *I* represents financial support, *T* spiritual support, and ω the saturation value of the support provided by children under the given constraints. *P*_*x*_ represents the total price of the goods and *x* the total number of goods, and *q* the quality of spiritual support provided by the children, which was assumed to be homogeneous in this study. *t* represents the amount (or time) of spiritual companionship provided by the children. The SWB of the parents left behind was represented using a CD (Cobb-Douglas) utility function. A deformation of Equations (1)–(4) yields the following:


(5) $$\begin{array}{l} U={\left(\frac{I}{P_{x}}\right)}^{\alpha }\frac{{\left(w-mI^{2}\right)}^{\frac{\beta }{2}}}{q^{\beta }n^{\frac{\beta }{2}}} \end{array}$$

Here, *U* is related to the magnitude of α and β, which was used to measure the preference of parents left behind for material goods vs. spiritual companionship. Thus, we hypothesized that the SWB of parents left behind depended on their preferences for the different types of support their children provided. Preferences were strongly individually heterogeneous; thus, α and β could vary accordingly after delineating parents left behind according to criteria.

This leads us to propose the following three hypotheses for verification:

H1: Children's internal migration has a significant positive effect on the subjective wellbeing of parents left behind.
H2: Children's internal migration has a significant negative effect on the subjective wellbeing of parents left behind.
H3: Financial support from children's migration has a positive effect on the subjective wellbeing of parents left behind, while reduced spiritual support has a negative effect through intergenerational transfer.
H4: There is heterogeneity in the direction of the total effect according to the preferences of parents left behind.

### 3.2. Econometric models

In this study, the explained variable SWB was a ranked discrete variable, and the core explanatory variable was a binary variable; therefore, the ordered logit (Ologit) model was chosen for estimation. For the purposes of this study, 2 years of microdata were selected for estimation. For the endogeneity problem caused by omitted variables, dummy variables for the province and year were introduced to construct the Ologit two-way fixed effect models for estimation. The model expression is as follows:


(6) $$\begin{array}{lll} Y^{*} & = & \log\left[\frac{Y\leq j|X_{it}}{1-\left(Y\leq j|X_{it}\right)}\right]=\beta X_{it}+\theta Z_{it}+\alpha_{i}+\tau_{i}+\varepsilon_{it} \end{array}$$

(7) $$Y\;=\{\begin{array}{c} 0,\;if\;Y^{*}\;<r_{1}\\ 1,\;if\;r_{1}<Y^{*}<r_{2}\\ j,\;if\;r_{j}<Y^{*}<r_{j+1}\\ \ldots \ldots \\ 10,\;if\;Y^{*}\geq r_{10} \end{array}$$

where Y^*^ is the unobservable latent variable determined by the explanatory variable with other control variables. *X*_*it*_ represents a vector of observations of the core explanatory variables. *Z*_*it*_ represents a vector of observations of other control variables. α_*i*_ represents province effects that do not vary over time, and τ_*i*_ the year effect. *i* = 1, 2…, *n* is the number of observations. ε_*i*_ is the random disturbance term. *Y* is the explained variable, which is an observed variable. *r*_1_, *r*_2_, …, *r*_10_ is the interval cutoff of the explained variable.

The coefficients estimated in the Ologit model β could not measure the change in Y with X, and further calculations of the marginal effects are needed. In this study, we referred to the marginal effects model of Huang et al. (34), which calculates how changes in the core explanatory variable *X* affect the probability of *Y* taking each value. The model is as follows:


(8) $$\begin{array}{l} \omega ={\frac{\partial P\left(y=i|x_{it}\right)}{\partial x_{it}}|}_{z=\bar{z}}\left(i=0,1,\ldots ,10\right) \end{array}$$

where ω measures the probability that when the core explanatory variable *X*_*it*_ changes by one unit, the change in the probability of the explained variable *Y* taking the value *i* when the other control variables are constant. The economic interpretation is how much higher (lower) the probability of SWB taking a value of *i* is for parents left behind with children's internal migration than for parents without children's internal migration.

## 4. Results

### 4.1. Descriptive statistics

Table 1 provides the descriptive statistics of the main characteristics of the study variables. After excluding the missing or invalid values of the control variables, the final valid sample size was 12,927. The mean value of the sample SWB was 7.68 and the median value was 8, indicating that the level of self-rated SWB in China is high. The sample size of children's internal migration accounted for 39.7% of the total sample size, which is also consistent with realistic experience, meaning the sample is representative. Among the control variables, the mean value of gender was about 0.5, the mean age of parents left behind was 67.95, access to education was generally poor, the mean values of self-rated health and self-rated socioeconomic status were both around the median, and the sample was more evenly distributed. The married sample accounted for 84.9% of the total sample, and the divorced and widowed sample accounted for 14.5%. The sample size for agricultural households was slightly larger than that for non-agricultural households. The sample contained a relatively small group with income from work in the past 12 months, although this was very low. The per capita consumption expenditure of households in 2019 was 11,630, which is lower than the national value of 19,853 per capita consumption expenditure of urban and rural residents according to the *China Statistical Yearbook 2019*. However, this may be influenced by older people's traditional view of consumption, namely that they prefer saving to consumption. Statistical descriptions from the perspective of parents left behind, as Table 1 shows, indicate that both the personal income and per capita household expenditure of parents left behind in households with children's internal migration are smaller than those in households without children's internal migration. This suggests that the economic situation and quality of life of households with children's internal migration are generally not high, which may encourage children to migrate for higher financial returns.

**Table 1:** Descriptive statistics of variables.

**Variable**	**Obs**	**Mean**	**Std**	**Min**	**Max**
SWB	12,927	7.678	2.206	0	10
Migration	12,927	0.397	0.489	0	1
Gender	12,927	0.506	0.500	0	1
Age	12,927	67.945	6.288	60	98
Marriage	12,927	2.426	1.040	1	5
Education	12,927	1.945	1.129	1	7
Registration	12,927	0.690	0.477	0	1
Health status	12,927	3.526	1.211	1	5
Social status	12,927	3.250	1.148	1	5
Personal income	12,927	1,066.045	6,110.448	0	120,000
Per capita household expenditure	12,927	11,630.463	21,770.935	0	900,000
**Migration** = **1**			**Migration** = **0**		
**Variable**	**Obs**	**Mean**	**Variable**	**Obs**	**Mean**
Personal income	5,130	447.052	Inc	7,797	1,474.540
Per capita household expenditure	5,130	10,628.020	Fexp	7,797	12,292.360

### 4.2. Ordered logit regression results

To verify the direction of the total effect of children's internal migration on the SWB of parents left behind, a regression analysis was performed on the model (6). A stepwise regression was used to ensure the robustness of the results. As Table 2 shows, children's internal migration has a significant negative effect on parents left behind through intergenerational transfer. As such, H2 is verified. Gender, age, marital status, type of household registration, health status, social status, and household expenditure per capita all had significant effects on the SWB of the parents left behind.

**Table 2:** Baseline regression results.

	**(1) SWB**	**(2) SWB**	**(3) SWB**
Migration	−0.183^***^	−0.111^***^	−0.101^**^
	(0.039)	(0.038)	(0.044)
Gender		−0.221^***^	−0.180^***^
		(0.045)	(0.044)
Age		0.026^***^	0.026^***^
		(0.004)	(0.004)
Marriage		−0.097^***^	−0.090^***^
		(0.021)	(0.021)
Education		−0.000	−0.034
		(0.021)	(0.021)
Household registration		−0.381^***^	−0.392^***^
		(0.050)	(0.053)
Health status		−0.273^***^	−0.256^***^
		(0.017)	(0.017)
Social status		0.330^***^	0.331^***^
		(0.018)	(0.018)
Personal income		−0.000	−0.000
		(0.000)	(0.000)
Per capita household expenditure		0.000	0.000^**^
		(0.000)	(0.000)
Year	No	No	Yes
Province	No	No	Yes
Cons	1.847^***^	1.358^***^	1.240^***^
	(0.117)	(0.101)	(0.097)
N	12,927	12,927	12,927
Lr chi^2^	540.76^***^	336.55^***^	294.34^***^

^***^, ^**^, and ^*^indicate significance at 1%, 5%, and 10%, respectively, standard errors are presented in parentheses.

### 4.3. Marginal effect

The marginal effect of the core explanatory variable “children's internal migration” on the SWB of parents left behind was calculated according to model (8). As Table 3 shows, at the 5% significance level, the likelihood that parents left behind with children's internal migration takes a value of 0, 1, 2, 3, 4, 5, 6, 7, and 8 is 0.073, 0.050, 0.096, 0.166, 0.144, 0.791, 0.262, 0.202, and 0.030% higher than parents without children's migration. Furthermore, children's internal migration is 0.178 and 1.636% less likely to make their parents left behind taking values of 9 and 10, respectively. In other words, children's internal migration makes it more likely that parents' SWB will take lower values and less likely that taking higher values, which is consistent with the direction of the total effect derived from the regression results in Table 3.

**Table 3:** Marginal effects of core explanatory variables.

**SWB**	**0**	**1**	**2**	**3**	**4**	**5**
Marginal effect	0.0007292^**^	0.0005032^**^	0.0009639^**^	0.0016624^**^	0.0014442^**^	0.0079116^**^
**SWB**	**6**	**7**	**8**	**9**	**10**	
Marginal effect	0.0026206^**^	0.00202^**^	0.0002962^**^	−0.0017869^**^	−0.0163643^**^	

^***^, ^**^, and ^*^indicate significance at 1%, 5%, and 10%, respectively.

### 4.4. Intermediary mechanism

We dissected the intrinsic mechanisms through which children's internal migration affects the SWB of parents left behind from the perspective of intergenerational transfer and examined the spiritual and financial support provided by children to their parents as two influential mechanisms.

Intergenerational spiritual support was measured by summing the values of “frequency of meeting” and “frequency of contact” in the CFPS, with higher values representing more spiritual support. Intergenerational financial support was measured according to the amount of “money sent home” (remittance) in the CFPS, with higher amounts representing more intergenerational financial support.

Table 4 provides the results of the mechanism test using a three-step approach, where children's internal migration has a negative effect on intergenerational spiritual support at the 1% significance level and spiritual support has a positive effect on the SWB of parents left behind at the 1% significance level. Similarly, children's internal migration has a positive effect on intergenerational financial support at the 1% significance level, and financial support has a positive effect on the SWB of parents left behind at the 1% significance level. Combined with the results of the baseline regression, the existence of two influential mechanisms, namely intergenerational spiritual and intergenerational financial support, was affirmed. Thus, H3 is verified.

**Table 4:** Results of the mechanism test.

	**Spiritual support**	**SWB**	**Financial support**	**SWB**
Migration	−0.327^***^		0.827^***^	
	(0.047)		(0.029)	
Spiritual support		0.073^***^		
		(0.007)		
Financial support				0.043^***^
				(0.013)

^***^, ^**^, and ^*^indicate significance at 1%, 5%, and 10%, respectively, standard errors are presented in parentheses.

To further determine the effect of these two mechanisms on the total effect, we used the KHB test to decompose the indirect effects of spiritual and financial support from the total effect. The KHB test controlled for control variables as well as year and province effects. Table 5 provides the results of the KHB test. The first column represents the total effect under the full model; the second column, the direct effect under the simplified model; the third column, the decomposed indirect effect; and the fourth column, the confounding percentage, which is the ratio of the indirect effect to the total effect. Table 5 shows that the indirect effect of spiritual support is 38.77%, while the direct effect is not significant. This means that spiritual support plays a major mediating role in the influence of the mechanism. (Since the significance of the direct effect is affected by the standard error, and expanding the sample size may enhance its significance and lead to the conclusion of partial mediation, we consider that full mediation does not imply sole mediation, which is interpreted here as “primary mediation”). The indirect effect of intergenerational financial support has a ratio of 51.07% and is in the opposite direction of the direct effect. This implies that financial support plays a masking effect rather than mediating effect in the mechanism impact. That is, children's internal migration reduces parents' SWB by reducing intergenerational spiritual support, but the intergenerational financial support that comes from children mitigates this negative effect.

**Table 5:** KHB test results.

	**Reduced**	**Full**	**Diff**	**Ratio**
Spiritual support	−0.087^**^	−0.053	−0.034^***^	38.77%
	(0.037)	(0.037)	(0.005)	
Financial support	−0.086^**^	−0.131^***^	0.044^***^	−51.07%
	(0.037)	(0.038)	(0.010)	

^***^, ^**^, and ^*^indicate significance at 1%, 5%, and 10%, respectively, standard errors are presented in parentheses.

### 4.5. Heterogeneity test

As mentioned in the theoretical model section, the preference for spiritual vs. financial support could subsequently differ after delineating the parents left behind into groups according to certain criteria. Thus, we explored the heterogeneity of the total effect and of the masking effect due to financial support based on the sub-sample.

The sample was delineated as high-income families (per capita income above 12,920) and low-income families (per capita income of 12,920 and below) based on the median per capita net household income of 12,920. The sample was also divided into an older parents group (over 67 years old) and a middle-aged parents group (67 years or younger) based on the median age of 67 years. Furthermore, categories included a high-level health group (self-rated health score above 3) vs. a low-level health group (self-rated health score 3 and below) according to median health level 3, and a middle and highly educated group (highest education value above 2) vs. a group with low levels of education (highest education 2 and below) according to median education level 2.

A split-sample regression analysis of the model (6) was first conducted to explore the heterogeneity of the total effect and then to separate the direct and indirect effects based on the total effect. Table 6 provides the Ologit regression results and Table 7 the KHB test results. There is heterogeneity in the direction of the total effect after dividing parents left behind according to the abovementioned criteria. Thus, H4 is verified.

**Table 6:** Sub-sample regression results.

	**Household income**		**Age**		**Health**		**Education**	
	>12,920	≤ 12,920	>67	≤ 67	>3	≤ 3	>2	≤ 2
Migration	−0.207^***^	−0.046	−0.144^**^	−0.066	−0.179^***^	−0.034	−0.200^**^	−0.072
	(−0.068)	(−0.059)	(−0.072)	(−0.057)	(−0.062)	(−0.063)	(−0.089)	(−0.051)

^***^, ^**^, and ^*^indicate significance at 1%, 5%, and 10%, respectively, standard errors are presented in parentheses.

**Table 7:** Sub-sample KHB test results.

	**High income**		**Aged parent**		**High health level**		**Highly educated**	
	**Spiritual support**	**Financial support**	**Spiritual support**	**Financial support**	**Spiritual support**	**Financial support**	**Spiritual support**	**Financial support**
Reduced	−0.186^***^	−0.186^***^	−0.119^**^	−0.115^*^	−0.145^***^	−0.142^***^	−0.187^***^	−0.186^***^
	(−0.055)	(−0.055)	(−0.060)	(−0.060)	(−0.053)	(−0.053)	(−0.073)	(−0.073)
Full	−0.142^**^	−0.228^***^	−0.074	−0.158^**^	−0.099^*^	−0.178^***^	−0.158^**^	−0.199^***^
	(−0.056)	(−0.057)	(−0.060)	(−0.062)	(−0.053)	(−0.054)	(−0.073)	(−0.075)
Diff	−0.044^***^	0.042^***^	−0.045^***^	0.043^***^	−0.047^***^	0.036^***^	−0.029^***^	0.013
	(−0.008)	(−0.015)	(−0.009)	(−0.015)	(−0.008)	(−0.013)	(−0.008)	(−0.020)
Ratio	23.53%	−22.83%	37.91%	−37.11%	32.10%	−24.93%	15.47%	−7.06%

^***^, ^**^, and ^*^indicate significance at 1%, 5%, and 10%, respectively, standard errors are presented in parentheses.

Children's internal migration has a significant negative effect on the SWB of parents left behind in high-income families, but no significant effect on low-income families. As shown in columns (1) and (2) of Table 8, after changing the sample distribution, spiritual support has a partial mediating effect on the SWB of parents left behind in high-income families at 23.53% when the masking effect from financial support is −22.83%. This indicates that not only is the total effect negative, but the masking effect from financial support does not fully offset the mediating effect of spiritual support.

**Table 8:** Robustness regression results.

	**SWB**	**SWB**	**SWB**
	**(1)**	**(2)**	**(3)**
Migration	−0.111^**^	−0.191^***^	−0.101^**^
	(0.046)	(0.059)	(0.043)
Gender	−0.176^***^	−0.205^***^	−0.136^***^
	(0.047)	(0.047)	(0.043)
Age	0.028^***^	0.017^***^	0.025^***^
	(0.004)	(0.004)	(0.003)
Marriage	−0.102^***^	−0.086^***^	−0.101^***^
	(0.022)	(0.023)	(0.020)
Education	−0.019	−0.016	0.007
	(0.023)	(0.022)	(0.021)
Household registration	−0.420^***^	−0.286^***^	−0.413^***^
	(0.057)	(0.055)	(0.052)
Health status	−0.258^***^	−0.248^***^	−0.238^***^
	(0.018)	(0.019)	(0.016)
Social status	0.333^***^	0.393^***^	0.296^***^
	(0.019)	(0.021)	(0.017)
Personal income	−0.000	−0.000	−0.000
	(0.000)	(0.000)	(0.000)
Per capita household expenditure	0.000^***^	0.000^**^	0.000^**^
	(0.000)	(0.000)	(0.000)

^***^, ^**^, and ^*^indicate significance at 1%, 5%, and 10%, respectively, standard errors are presented in parentheses.

Children's internal migration has a significant negative effect on the SWB of older parents, but no significant effect on middle-aged parents. As shown in columns (3) and (4) of Table 8, children's internal migration has a negative effect on parents' SWB, mainly due to reducing spiritual support to parents, although intergenerational financial support mitigates part of the negative effect, masking the effect by −37.11%, but not enough to change the direction of the total effect.

Children's internal migration has a significant negative effect on the SWB of parents with a high level of health, but no significant effect on parents with a low level of health. As shown in columns (5) and (6) of Table 8, children's internal migration has a negative effect on the SWB of parents with high health through spiritual support, with a mediating effect of 32.10% and a masking effect of financial support of −24.93%.

Finally, children's internal migration has a significant negative effect on the SWB of middle and highly-educated parents but no significant effect on those with a low level of education. As shown in columns (7) and (8) of Table 8, the partial mediating effect of spiritual support on the SWB of parents left behind in families with high education is 15.47%, while the indirect effect of financial support on parents' SWB is no longer significant.

### 4.6. Robustness check

We performed robustness tests in the following three areas:

(1) Adjusting the classification of the explained variable: There are 11 levels of SWB evaluation in the CFPS. In this study, the original levels were combined and reassigned as follows: 0–2 assigned 1, 3–4 assigned 2, 5–6 assigned 3, 7–8 assigned 4, 9–10 assigned 5, and then an Ologit two-way fixed effects model was constructed for the regression analysis.
(2) Changing the sample data: The data used above is the panel data consisting of CFPS2018 and CFPS2016. In this study, the sample was reduced to CFPS2018 cross-sectional data, and then an Ologit model was constructed for the regression analysis.
(3) Changing the model and test method: A two-way fixed effects model with panel data was constructed to test the above findings, based on which the Sobel and Bootstrap tests were performed to verify the mechanism of the influence of moral and financial support.

Table 8 provides the results of the Ologit regression for the robustness test. Regardless of adjusting the classification of the explained variable, changing the sample data, or changing the model and test method, children's internal migration has a significant negative effect on the SWB of parents left behind. Thus, our conclusion was robust regarding the direction of the total effect. Table 9 provides the results of the influence mechanisms under the robustness test: the bootstrap 95% interval does not contain 0 for either spiritual or financial support, affirming the existence of these two influence mechanisms. The results of the Sobel test show that the direct effect of spiritual support is not significant. The masking effect from financial support is significant at the 1% level and accounts for −46.01% of the total effect. Thus, children's internal migration had a negative effect on parents' SWB mainly through reduced spiritual support, and financial support mitigated this negative effect. This finding is consistent with that mentioned above.

**Table 9:** Sobel vs. bootstrap test results.

**Variable**	**Coefficient A**	**Coefficient B**	**Total effect**	**Direct effect**	**Indirect effect**	**Indirect effects ratio**	**Bootstrap 95% confidence interval**
Spiritual support	−0.522^***^	0.081^***^	−0.105^**^	−0.063	−0.042^***^	40.01%	(−0.054, −0.031)
Financial support	0.893^***^	0.054^***^	−0.105^**^	−0.015^***^	0.048^***^	−46.09%	(0.026, 0.071)

^***^, ^**^, and ^*^indicate significance at 1%, 5%, and 10%, respectively, standard errors are presented in parentheses.

## 5. Discussion

Our empirical study revealed that children's internal migration has an overall negative effect on the SWB of parents left behind, suggesting that older people have stronger preferences for factors such as daily care, caring and love, and spiritual support relative to factors such as increased economic power and improved social status brought about by children's internal migration. Thus, the SWB of parents left behind significantly decreased when children's internal migration led to greater spatial distance and therefore, less spiritual support for the parents. Our results are consistent with those of previous studies (21, 40).

Furthermore, we validated two mechanisms of intergenerational social support: financial and spiritual support (41). Higher intergenerational financial support enhances parents' SWB as a result of higher economic rewards from children's internal migration (42). Children's internal migration separates families, the busy work schedule and increased geographical distance reduce the time spent by children with their parents, and the reduced frequency of parent-child contact reduces parents' SWB (43). To conclude, children's internal migration reduced parents' SWB by reducing intergenerational spiritual support, but the intergenerational financial support brought about by migration mitigated this negative effect.

Delineating the group of parents left behind according to per capita net household income, age, health status, and education, the effect sizes of the two types of social support varied, validating the hypotheses derived from the theoretical model. The empirical results of the sub-sample divided according to net per capita household income indicated no significant effect of children's internal migration on low-income families. Low-income families are generally worse off economically, and the economic gains from migration compensate for the “emotional side effects” of the estrangement between children and their parents (44). Thus, the total effect is not significant, while the preference for intergenerational financial support is relatively lower among older adults in higher-income families. As such, the financial support provided after children's internal migration has a limited mitigating effect on parents' SWB. The finding that income level affects the extent of the association between remittances and SWB is consistent with previous findings (39).

The empirical results of the sub-sample delineated according to age indicated no significant effect of children's internal migration on middle-aged parents. Middle-aged parents have relatively strong connections to others in their social networks (45). Active social engagement with sources of social support such as friends and neighbors can help parents cope with emotions such as loneliness and isolation (46). They, therefore, have a relatively low need for spiritual support from their children. However, older parents have a higher preference for spiritual support from their children as their social networks become increasingly narrower, meaning that companionship and care from their children are crucial.

The empirical results of the sub-sample divided according to health status indicated no significant effect of children's internal migration on parents with a low level of health. The quantity and quality of healthcare investments required by parents with a low level of health are higher. Here, the positive impact of remittances on the share of the household health budget (5, 47) and on improving the health of parents left behind (48) is evident. This increases parents' preference for financial support, which ultimately does not differ significantly from their preference for spiritual companionship. Thus, the total effect is not significant. On the other hand, parents in better health have a relatively weaker preference for intergenerational financial support and a relatively stronger preference for spiritual support.

The empirical results of the sub-sample delineated according to education indicated no significant effect of children's internal migration on parents with low education. There are two possible reasons the response of highly educated parents to financial support is not significant. First, because the returns to education are positive (49), the preference of highly educated parents for financial support is similar to that of parents with high-income levels. Second, highly educated parents tend to have high spiritual intelligence (50) with a greater focus on communication in the spiritual world as opposed to material goods. To test this conjecture, we used a three-step approach, which showed that children's internal migration significantly enhanced financial support. However, that financial support had no significant effect on the SWB of highly educated parents.

Our findings are robust in terms of different classifications of SWB, sample data selection, models, and test methods.

Our study extends beyond the existing literature in the following aspects. First, we focused on the effect of internal migration, which is much higher than international migration for a country with a large population like China. As noted, most of the literature focuses on international migration. Second, we focused on the SWB of the parents left behind, which is aligned with the trend of an aging population. As mentioned, most of the literature focuses on the mental health of the children left behind. Third, spiritual and financial support are two important channels of intergenerational social support. We separated and compared their influential roles and further examined the heterogeneity of parents' preferences for these two dimensions of social support across different groups, linking parental characteristics to social support preferences.

However, our research has some limitations. First, this study only separates and compares two dimensions of intergenerational social support, but the positive effect of children's internal migration on the SWB of the parents left behind is not limited to financial support and the negative effect is not limited to spiritual support. Thus, a further literature analysis could be considered in the future to identify more dimensions of preferences of older adults. Second, causality should be interpreted with caution when considering issues of endogeneity. Finally, the conclusions may not apply to every country because of differences in national circumstances.

## 6. Conclusion

In this study, we empirically examined the impact of children's internal migration on the SWB of parents left behind based on population mobility trends and the aging context. To this end, we employed panel data from the CFPS2016 and CFPS2018. First, we found that children's internal migration had a significant negative effect on the SWB of parents left behind. Second, children's internal migration reduced the SWB of parents left behind by reducing spiritual support, and the increase in intergenerational financial support brought about by the internal migration had a significant mitigating effect on this negative effect. Third, parents with different characteristics had different preferences for positive and negative outcomes brought about by children's internal migration. Fourth, parents' preferences for spiritual and financial support differed across characteristics, so while there was heterogeneity in the size of the masking effect of financial support, this masking effect could never fully offset the negative effect of reduced spiritual support on parents' SWB.

To address the negative effects of children's internal migration on parents, positive measures should be taken to change parental preferences.

The first measure is to reduce the dependence of parents left behind on their children for spiritual support. By improving policy measures such as employment, volunteering, and community governance, older people physically able to actively participate in society should be encouraged to provide social support to others (51). Parents should also be encouraged to actively participate in social and recreational activities. To this end, cultural activities should be organized, and diverse services and environments provided for performances and events (52). Next, opportunities and conditions for parents left behind should be created to enable them to make friends through various activities, strengthen their ties with others in social networks to meet social needs, and gain opportunities to communicate with others and express their emotions (53).

Second, the integration of family and social aging should be promoted. Businesses and industries catering to the needs and care of older adults should be developed, services for older adults should be optimized, and basic care services for all older people should be promoted and established. The pension insurance system must be improved, the pension replacement rate increased, and the quality of life of parents left behind guaranteed (54). Finally, the level of care services for the older population needs to be improved to meet their multi-level and diversified needs and to enrich their spiritual life in addition to their material needs. The subjective wellbeing of parents left behind can be improved by improving the social security and elderly care service systems.

## Data availability statement

Publicly available datasets were analyzed in this study. This data can be found here: http://www.isss.pku.edu.cn/cfps.

## Ethics statement

The studies involving human participants were reviewed and approved by Biomedical Ethics Committee of Peking University. Written informed consent to participate in this study was provided by the participants' legal guardian/next of kin.

## Author contributions

YL and YZ conceived this research. YL was responsible for the methodology and software, conducted the formal analysis, and wrote the original draft. Y-YL was responsible for the investigation and was in charge of review and editing. J-QQ was responsible for data curation. YZ was in charge of conceptualization and acquired resources and supervised the project administration. W-ZW conducted the validation. All authors contributed to and have approved the final manuscript.

## Footnotes

^1^Data source: The Seventh National Population Census.

^2^Note that the positive impact is never just an increase in financial support, and the negative impact is never just a lack of spiritual support; the model is simplified here only for the sake of better analysis.
